# Estimating Socioeconomic Status for Health Equity Surveillance in Cameroon: An Expert Opinion Survey

**DOI:** 10.7759/cureus.116688

**Published:** 2026-09-21

**Authors:** Collins Buh Nkum, Yannick Niamsi Emalio, Marie Nicole Ngoufack, Gilles Protais Lekelem Dongmo, Brian Bongwong Tamfon, Aude Nanfak, Jerome Sedowo Eyi Kodjo, Alain Chichom-Mefire, Catherine Juillard, Alan Hubbard, Georges Nguefack-Tsague

**Affiliations:** 1 Department of Health Research, Meilleur Accès aux Soins de Santé, Yaounde, CMR; 2 Department of Public Health, University of Yaounde 1, Yaounde, CMR; 3 Department of Data Science, Higher Institute for Scientific and Medical Research, Yaounde, CMR; 4 Department of Chemistry-Biology, University of Bertoua, Bertoua, CMR; 5 Department of Cardiology, Vrije Universiteit Brussel, Brussels, BEL; 6 Division of Health Research, Challenges Initiative Solutions, Yaounde, CMR; 7 Division of Health Research, Challenges Initiative Solutions, Yaoundé, CMR; 8 Department of Surgery, University of Buea, Buea, CMR; 9 Department of Surgery, University of California Los Angeles, Los Angeles, USA; 10 Division of Biostatistics, University of California Berkeley, Berkeley, USA

**Keywords:** demographics and health survey, expert opinion, health equity, prior probability, socioeconomic status

## Abstract

Background: Despite growing awareness of the association between socioeconomic status (SES) and health outcomes, no widely accepted, rapidly implementable method for estimating SES exists in resource-limited settings. An exception is the Demographic and Health Surveys (DHS) wealth quintile index constructed from household asset ownership. To support health equity surveillance, a method of individual SES estimation that requires fewer household assets is needed. This study aimed to identify the DHS household asset items most relevant for measuring SES in Cameroon. Materials and methods: This cross-sectional study assessed expert opinion on the indicators used to measure SES. Participants were interviewed using a structured questionnaire and included stakeholders involved in designing and implementing DHS in Cameroon for many years. Using a five-point Likert scale, experts rated the relevance of DHS household asset items for measuring SES. The questionnaire was highly reliable (Cronbach’s alpha: 0.943, 95% CI: 0.920-0.961, p < 0.001) for the 29 retained items measuring SES. Results: Among the 65 experts with complete responses, the proportion agreeing or strongly agreeing that an asset is a useful measure of SES ranged from 29.2% to 84.6% across the 29 items; a normalized weight (each item's share of the total agreement counts across items, summing to 1) was also derived for each item and ranged from 0.016 to 0.047. The 12 DHS assets with the highest normalized weight included refrigerator (84.6%), television (83.1%), laptop/notebook (80.0%), agricultural land (80.0%), mixer/grinder (78.5%), cable/satellite (78.5%), cell phone (76.9%), modem/Internet key (75.4%), computer tablet (73.8%), gas stove (72.3%), car/truck (72.3%), and water pump (70.8%), with respective normalized weights of 0.047, 0.046, 0.044, 0.044, 0.043, 0.043, 0.042, 0.042, 0.041, 0.040, 0.040, and 0.039.

Conclusions: This research underscores the importance of integrating local expert insights to refine SES measurement and improve health outcomes, particularly in Cameroon. Future research should explore applying this expert-opinion-driven framework in various contexts to create more comprehensive, robust, and reliable SES indicators.

## Introduction

Despite growing awareness of the association between socioeconomic status (SES) and health outcomes, no widely accepted, readily implementable SES measure exists for resource-limited settings [1,2]. Health inequities are particularly unmasked by acute conditions, such as trauma, where low SES is both a risk factor for injury and associated with poor outcomes. The Demographic and Health Surveys (DHS) wealth quintile index is constructed from household asset ownership, limiting data collection in time-sensitive clinical conditions such as trauma. To support health equity surveillance in acute care settings, we need an individual SES estimation method that requires fewer household assets. Since the 1980s, nations around the world have been increasingly called upon to address health inequities, which are systematic, unfair, and avoidable differences in health outcomes and their determinants between segments of the population, such as SES, demography, or geography [3-5]. Health equity represents the absence of systematic health disparities between groups with different economic statuses [1]. Because societies are not organized equitably, opportunities to thrive and be healthy are not distributed equally between and within societies. For example, people in low-income countries are much more likely to die or become disabled as a result of trauma because of the high cost of treatment, which is often surgical [6,7]. Evaluating public health equity is important to ensure interventions do not increase health inequalities and meet their stated objectives to reduce health inequities.

Monitoring health inequities and measuring the impact of interventions have been identified by the World Health Organization (WHO) as key strategies needed to address disparities [8]. This monitoring requires understanding the SES of users seeking healthcare. A major obstacle to addressing equity issues in resource-limited settings is the lack of population-specific, validated, and easily implemented SES measures applicable across contexts. One available approach is the WHO-provided method, which uses DHS-derived data to establish wealth quintiles [9]. Several tools, still incorporating DHS data, have been designed to improve the WHO tool [10]. However, these measures have limitations: they do not collect individual-level wealth data and therefore do not address inequality, as they are not designed to monitor individual-level SES precisely through primary data collection in the field. In Cameroon and other countries, researchers use factors such as education, occupation, income, and some of the 29 DHS household asset indicators to characterize an individual's SES [11]. Income, although a reliable reflection of economic status, can be difficult to assess in low-income countries where employment is generally irregular [12]. Assessing consumption and expenditure is even more time-consuming in acute care settings, where time and patient tolerance are limited. The lack of quick and easy-to-implement tools to accurately measure SES in acute care settings remains a significant barrier to monitoring and mitigating health disparities in trauma and other time-sensitive conditions.

Determining a patient's SES requires healthcare staff to collect data on the 29 indicators used in the measurement of the SES to obtain a classification of the patient's level according to the wealth quintile, which may not be feasible in acute care settings because the management must be done within a short time to improve patient outcomes. When patients arrive in hospital with an illness, it is difficult to assess a patient's well-being quintile by completing the 29 DHS indicators [13,14]. Completing all 29 indicators takes more time and therefore increases the risk of negative health consequences for the patient admitted to hospital. In this case, it is harder to determine SES and whether the patient has certain advantages.

Various methods, including statistical and mathematical techniques, exist to reduce the number of variables. For instance, the economic cluster is an analysis-based algorithm that reduces variables by selecting four or five from 29 DHS metrics [15,16]. This algorithm uses simple, population-specific SES measures from nationally representative DHS household-item data [17]. This technique relies exclusively on statistical methods and has not yet been validated by experts in survey data collection and analysis in Cameroon. To ensure complementarity, we propose capturing, in parallel, the main variables to retain based on expert opinions in the field. This study therefore had two objectives. First, we sought to identify which of the 29 DHS household asset indicators experts consider the most relevant measures of SES in Cameroon. Second, we aimed to quantify expert agreement for each item as prior probabilities, which can serve as expert-derived weights in future Bayesian or statistical modeling.

We previously posted this manuscript to the Research Square preprint server on January 2, 2025 [18].

## Materials and methods

Study design, period, and setting

This study used a cross-sectional design to assess expert opinion on indicators used to measure SES through an in-person interview. Expert opinion surveys are useful for creating rankings when more objective data are not available. Data were collected between December 21, 2023, and April 10, 2024, in Yaoundé (the capital of Cameroon), targeting experts from institutions and organizations involved in the design, implementation, or other aspects of the DHS in Cameroon for many years.

Study population and sampling

Experts were identified from DHS reports published online, which document the individuals and institutions involved in the design, implementation, and analysis of the DHS in Cameroon over the years. We targeted experts in the fields of Statistics, Economics, Demography, Social Sciences, and Health who had been involved in the DHS in Cameroon. An exhaustive sampling technique was used, whereby all eligible experts identified from these sources were invited to participate; this approach ensured that the sample encompassed a diverse range of professional backgrounds, including researchers, policymakers, and practitioners affiliated with universities, governmental bodies, and non-governmental organizations.

Eligibility criteria

For purposive sampling, an "expert" was operationally defined as a professional holding at least a bachelor's-equivalent qualification in a DHS-relevant discipline (e.g., statistics, demography, economics, or public health) and having a minimum of one year of documented, direct involvement in at least one stage of the design, implementation, or analysis of the Cameroon DHS (or an equivalent national household survey), such as questionnaire design, fieldwork or data collection, data management, or data analysis and reporting. Inclusion criteria were (1) professional or institutional affiliation involving direct participation in at least one stage of the design, implementation, or analysis of DHS (or an equivalent national household survey) in Cameroon, consistent with the operational definition of "expert" above; and (2) provision of informed consent to participate, following a comprehensive briefing on the study's objectives.

Exclusion criteria, independent of the inclusion criteria above, were (1) current membership of the study's research team or involvement in designing the survey instrument used in this study to avoid conflict of interest; (2) unavailability or inability to be reached for interview within the data-collection period; and (3) a questionnaire left incomplete such that eligibility could not be confirmed, or key responses were unusable.

Variables

Two distinct sets of variables were collected: (i) DHS SES items rated and (ii) expert characteristics. The first set comprised the 29 DHS household asset items that experts rated for their relevance as measures of SES, spanning four domains: durable goods (e.g., televisions, refrigerators), household infrastructure (e.g., roofing and wall materials), utilities (e.g., electricity and sanitation), and agricultural assets (e.g., land and livestock). The second set described the experts themselves, comprising (a) sociodemographic and professional characteristics: age, sex, marital status, professional/disciplinary background, highest level of education, years of professional experience, and type of institution; and (b) DHS-exposure characteristics: access to DHS data, training received on DHS, involvement in the design of DHS, and involvement in the analysis of DHS data.

Questionnaire development

The questionnaire was built around the 29 household asset items used to construct the standard DHS wealth index, with item wording adapted from the DHS Model Questionnaire and translated into French, the working language of the data collection team in Cameroon. Senior researchers experienced in DHS methodology reviewed the adapted instrument to assess content relevance and clarity of wording, and it was pretested with a small group of experts who were not part of the main study sample; feedback from this pretest informed minor revisions to item wording and response formatting before the final version was programmed into the Open Data Kit (ODK) platform used for data collection.

Data collection procedure and management

Experts were identified and invited to participate in the study. To administer the questionnaires, we used ODK. After data entry through ODK, we implemented comprehensive quality control measures to identify potential inaccuracies, such as numeric inconsistencies or missing values. Each questionnaire was rigorously double-checked for completeness and reliability.

Survey data analysis

The first step was to present the socio-demographic characteristics of the experts surveyed. This involved summarizing the data using frequencies, with the denominator for each variable restricted to experts with a non-missing response. Following this, the reliability of the questionnaire was assessed using the Cronbach's alpha coefficient (with 95% confidence interval (CI)) to ensure consistency among DHS asset items. For measuring expert opinions on the likelihood of DHS household assets to effectively represent SES, a 5-point Likert scale was utilized, enabling experts to indicate their level of agreement (i.e., strongly agree, agree, neither agree nor disagree, disagree, or strongly disagree) regarding each asset item. We recoded the 29 asset items into a binary variable: 1 for agree or strongly agree, and 0 for all other responses; this recoding was restricted to experts with complete responses across all 29 items. For each item, we report (i) the raw proportion of experts who agreed or strongly agreed and (ii) a normalized weight, calculated as that item's agreement count divided by the sum of agreement counts across all 29 items (so the 29 weights sum to 1). Data were collected, entered into ODK, and analyzed with R software version 4.3.0 (R Foundation for Statistical Computing, Vienna, Austria).

Ethical considerations

The study obtained ethical approval from the Centre Regional Ethics Committee for Human Health Research (CRERSH) (approval number: 00/57/CRERSHC/2023) and was conducted in accordance with the Declaration of Helsinki. Before administering each questionnaire, the surveyor explained the study's objectives and procedures to each expert using an information sheet and collected verbal and written consent from those willing to participate. The research team respected data anonymity and confidentiality throughout the process. The research team obtained informed consent from all participants.

## Results

Of the 70 experts identified and approached, 67 (95.7%) enrolled and completed the interview; the remaining three were not enrolled: two declined to participate, and one could not be reached during the data collection period. Table 1 presents the sociodemographic characteristics of the experts interviewed. We included 67 experts: 50 (74.6%) male and 17 (25.4%) female. Regarding marital status, 47% of the experts were married, and 47% were single. Professional/disciplinary backgrounds were concentrated in statistics and demography, with 35 participants (52.2%) and 21 participants (31.3%), respectively. Other fields, such as economics, medicine, and nutrition, were less represented. Work experience varied among participants, with notable representation across all categories: using the table's own categories, 32% of experts (21/66) reported "8 or more" years of experience, 27% (18/66) reported "6-7" years, 24% (16/66) reported "1-3" years, and 17% (11/66) reported "4-5" years (Table 1).

**Table 1:** Sociodemographic and professional characteristics of the experts n/N: denominators reflect non-missing responses for each variable; percentages are of non-missing responses. Total enrolled sample: N = 67.

Variable	Frequency, n/N (%)
Gender	
Female	17/67 (25)
Male	50/67 (75)
Age, mean (SD), years	33.7 (5.7)
Age group (years)	
25-29	14/61 (23)
30-34	26/61 (43)
35-39	14/61 (23)
40-59	7/61 (11)
Marital status	
Single	31/66 (47)
Cohabiting	3/66 (5)
Married	31/66 (47)
Widowed	1/66 (2)
Professional/disciplinary background	
Demographer	21/67 (31)
Economist	3/67 (4)
Mathematics	1/67 (1)
Nutrition	1/67 (1)
Reproductive health	1/67 (1)
Public health	2/67 (3)
Social sciences	1/67 (1)
Statistics	35/67 (52)
Medicine	2/67 (3)
Highest level of education	
Technician engineer	6/66 (9)
Conceptual engineer (bachelor equivalent)	16/66 (24)
Master's	38/66 (58)
Doctorate/PhD	6/66 (9)
Years of professional experience	
1-3	16/66 (24)
4-5	11/66 (17)
6-7	18/66 (27)
8 or more	21/66 (32)

Reliability of the questionnaires

The 29 items retained in the survey showed excellent internal consistency, with a Cronbach's alpha of 0.943 (95% CI: 0.920-0.961).

Participant exposure to DHS

All experts reported being familiar with the DHS (Figure 1).

**Figure 1:**
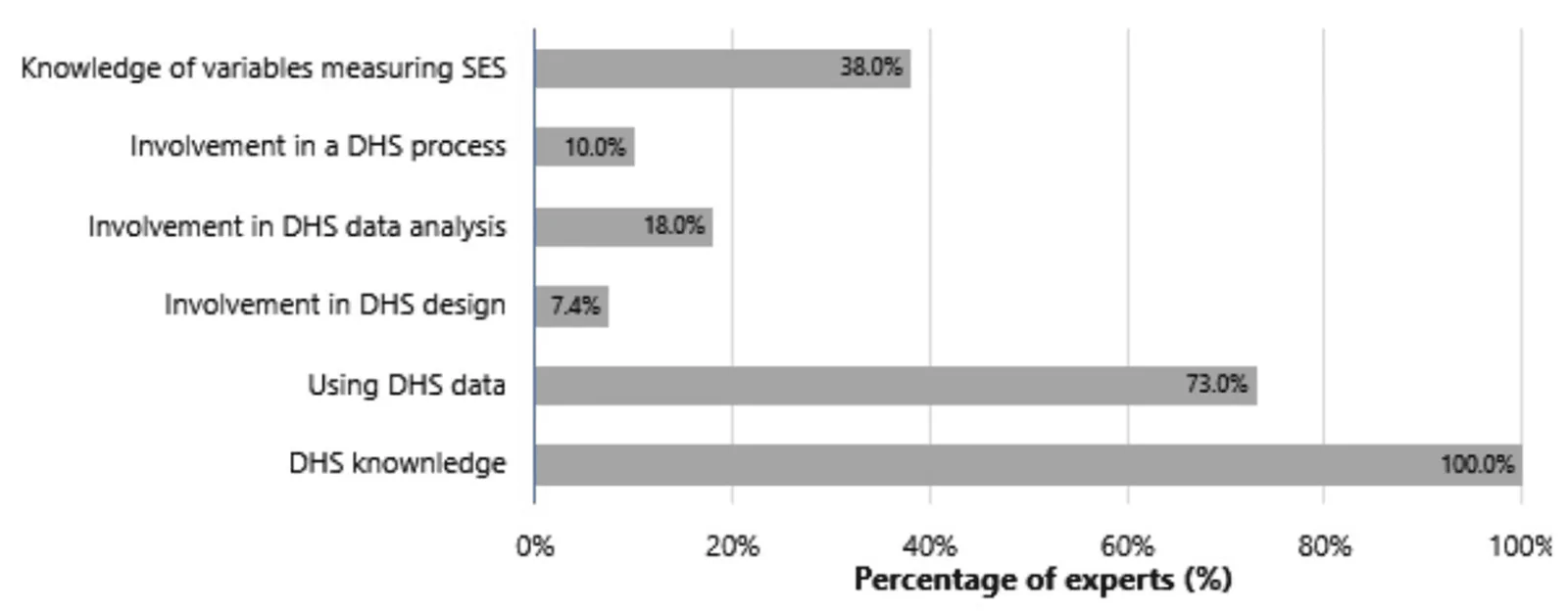
Participant exposure to DHS DHS: Demographic and Health Surveys, SES: socioeconomic status

Similarly, 73% of experts stated that they used DHS data. However, they were less involved in designing and implementing these surveys. Regarding the variables used in the DHS to measure SES, 38% of the respondents indicated that they were familiar with them.

Level of expert opinion on the 29 variables used to measure SES

Table 2 presents expert opinions on the 29 household asset indicators used to measure socioeconomic status. Respondents rated responses on a scale of 1 to 5 (1 = strongly disagree, 2 = disagree, 3 = neither agree nor disagree, 4 = agree, and 5 = strongly agree). Expert opinions varied across items and response categories. For television ownership, 52.3% agreed, and 30.8% strongly agreed that it was a useful measure of SES. Similarly, for cell phone ownership, 33.8% agreed, and 43.1% strongly agreed. Refrigerator ownership also received strong positive responses, with 43.1% agreeing and 41.5% strongly agreeing. In contrast, CD/DVD player ownership received lower levels of positive responses, with 18.5% agreeing and 10.8% strongly agreeing. Among transportation-related items, 38.5% agreed, and 33.8% strongly agreed that car/truck ownership was a useful measure of SES, whereas responses regarding animal-drawn cart ownership were more evenly distributed. Laptop/notebook and computer tablet ownership also received substantial positive responses, with 43.1% and 47.7% agreeing and 36.9% and 26.2% strongly agreeing, respectively.

**Table 2:** Distribution of expert Likert-scale ratings of the 29 DHS asset items (N = 65) Denominator N = 65 experts with complete responses across all 29 asset items (2 of the 67 enrolled experts had incomplete responses on this section and were excluded. DHS: Demographic and Health Surveys

Item	Strongly disagree	Disagree	Neither agree nor disagree	Agree	Strongly agree
Radio	6 (9.2)	14 (21.5)	11 (16.9)	25 (38.5)	9 (13.8)
Television	3 (4.6)	2 (3.1)	6 (9.2)	34 (52.3)	20 (30.8)
Home phone	12 (18.5)	10 (15.4)	9 (13.8)	18 (27.7)	16 (24.6)
Desktop computer	6 (9.2)	8 (12.3)	9 (13.8)	22 (33.8)	20 (30.8)
Refrigerator	3 (4.6)	3 (4.6)	4 (6.2)	28 (43.1)	27 (41.5)
Stove	3 (4.6)	6 (9.2)	11 (16.9)	21 (32.3)	24 (36.9)
Gas stove	1 (1.5)	6 (9.2)	11 (16.9)	26 (40.0)	21 (32.3)
Air conditioner	6 (9.2)	9 (13.8)	11 (16.9)	16 (24.6)	23 (35.4)
Fan	1 (1.5)	9 (13.8)	14 (21.5)	26 (40.0)	15 (23.1)
CD/DVD player	9 (13.8)	21 (32.3)	16 (24.6)	12 (18.5)	7 (10.8)
Grinding mill	6 (9.2)	15 (23.1)	16 (24.6)	24 (36.9)	4 (6.2)
Mixer/grinder	2 (3.1)	4 (6.2)	8 (12.3)	33 (50.8)	18 (27.7)
Modem/Internet key	2 (3.1)	6 (9.2)	8 (12.3)	26 (40.0)	23 (35.4)
Cable/satellite	2 (3.1)	4 (6.2)	8 (12.3)	28 (43.1)	23 (35.4)
Generator	8 (12.3)	3 (4.6)	13 (20.0)	17 (26.2)	24 (36.9)
Solar panel	6 (9.2)	3 (4.6)	14 (21.5)	16 (24.6)	26 (40.0)
Water pump	3 (4.6)	3 (4.6)	13 (20.0)	17 (26.2)	29 (44.6)
Clock/pendulum	7 (10.8)	13 (20.0)	16 (24.6)	23 (35.4)	6 (9.2)
Watch	6 (9.2)	16 (24.6)	13 (20.0)	23 (35.4)	7 (10.8)
Cell phone	3 (4.6)	5 (7.7)	7 (10.8)	22 (33.8)	28 (43.1)
Bicycle	6 (9.2)	14 (21.5)	21 (32.3)	15 (23.1)	9 (13.8)
Motorcycle/scooter	3 (4.6)	10 (15.4)	18 (27.7)	19 (29.2)	15 (23.1)
Animal-drawn cart	11 (16.9)	10 (15.4)	20 (30.8)	13 (20.0)	11 (16.9)
Car/truck	6 (9.2)	3 (4.6)	9 (13.8)	25 (38.5)	22 (33.8)
Motorboat	13 (20.0)	12 (18.5)	10 (15.4)	13 (20.0)	17 (26.2)
Laptop/notebook	1 (1.5)	6 (9.2)	6 (9.2)	28 (43.1)	24 (36.9)
Computer tablet	3 (4.6)	6 (9.2)	8 (12.3)	31 (47.7)	17 (26.2)
Agricultural land	2 (3.1)	4 (6.2)	7 (10.8)	27 (41.5)	25 (38.5)
Farm animals	4 (6.2)	10 (15.4)	12 (18.5)	23 (35.4)	16 (24.6)

Socioeconomic variables according to experts' preferences

We grouped each item into a binary variable, where 1 corresponds to agree or strongly agree responses and 0 to the other modalities. The raw proportion of experts who agreed or strongly agreed that an item measures SES ranged from 29.2% to 84.6% (Table 3). The items with the highest normalized weight were refrigerator (0.047), television (0.046), laptop/notebook and agricultural land (0.044 each), mixer/grinder and cable/satellite (0.043 each), cell phone (0.042), modem/Internet key (0.042), and computer tablet (0.041).

**Table 3:** DHS asset items according to experts’ agreement and normalized weight of each item (N = 65) * Each item's agreement count is divided by the sum of agreement counts across all 29 items. DHS: Demographic and Health Surveys

Item	Agree/strongly agree, n (%)	Normalized weight*
Refrigerator	55 (84.6)	0.047
Television	54 (83.1)	0.046
Laptop/notebook	52 (80.0)	0.044
Agricultural land	52 (80.0)	0.044
Mixer/grinder	51 (78.5)	0.043
Cable/satellite	51 (78.5)	0.043
Cell phone	50 (76.9)	0.043
Modem/Internet key	49 (75.4)	0.042
Computer tablet	48 (73.8)	0.041
Gas stove	47 (72.3)	0.040
Car/truck	47 (72.3)	0.040
Water pump	46 (70.8)	0.039
Stove	45 (69.2)	0.038
Desktop computer	42 (64.6)	0.036
Solar panel	42 (64.6)	0.036
Fan	41 (63.1)	0.035
Generator	41 (63.1)	0.035
Air conditioner	39 (60.0)	0.033
Farm animals	39 (60.0)	0.033
Radio	34 (52.3)	0.029
Home phone	34 (52.3)	0.029
Motorcycle/scooter	34 (52.3)	0.029
Watch	30 (46.2)	0.025
Motorboat	30 (46.2)	0.025
Clock/pendulum	29 (44.6)	0.025
Grinding mill	28 (43.1)	0.024
Bicycle	24 (36.9)	0.020
Animal-drawn cart	24 (36.9)	0.020
CD/DVD player	19 (29.2)	0.016

## Discussion

SES combines factors that affect an individual's or a family's health. These factors may include education, income, and employment type [19]. Measuring SES through indicators of goods ownership is a common approach in public health and social research. The DHS is a widely used survey tool that includes items on goods ownership to assess household wealth and SES. The DHS includes these items because they may directly influence health outcomes [12].

The most frequent approach to constructing an item index from a set of variables is a statistical method known as principal component analysis (PCA). PCA is a data-reduction technique that uses correlations among indicators to create a series of uncorrelated principal components [14,17,20]. This study aimed to propose an alternative to statistical and mathematical models based on expert opinion for selecting DHS variables used to measure SES and to obtain prior probabilities for each selected DHS item.

Selecting ownership-of-goods indicators can provide valuable insights into household living standards. Our findings indicate that the experts identified ownership of a refrigerator, television, laptop/notebook, mixer/grinder, and computer/tablet as the most important indicators in measuring SES. These results show clear consensus on certain items, such as refrigerators and televisions, which experts strongly agreed were critical SES indicators. This strong agreement highlights their perceived importance in creating a stable household environment, as they are often associated with basic needs and comfort. In contrast, items such as motorboats and CD/DVD players received lower agreement, suggesting that the relevance of certain items may be context-dependent and less aligned with current socioeconomic paradigms.

When examining the probabilities assigned to various items to measure SES, items such as refrigerators and televisions emerged as the most strongly supported, indicating their integral role in determining SES. The quantitative assessment offers a compelling argument for prioritizing specific items in future versions of the DHS. By focusing on high-agreement measures, policymakers and researchers can ensure that the most relevant indicators are incorporated into assessments of socioeconomic well-being, thereby improving the reliability and validity of the conclusions drawn.

Additionally, the prominence of technological items such as laptops and tablet computers among the selected variables reflects a wider socioeconomic trend towards digital inclusion. Access to technology is increasingly seen as a determinant of economic status, influencing opportunities for education, employment, and social mobility. This shift suggests that measures of SES must continually evolve, incorporating emerging item classes that respond to changing economic landscapes.

Other studies have suggested similar items to estimate SES and inequality within populations [14,21,22]. A related study used the Delphi method to develop shortened wealth indices using survey data from 16 countries; for Cameroon, it identified nine items, six of which (television, cable/satellite, refrigerator, fan, mixer/grinder, and watch) were asset-ownership indicators [10]. Similarly, a study in Iran concluded that six items out of 33 in a simple item index comprised kitchen, bathroom, vacuum cleaner, washing machine, freezer, and personal computer [23]; unlike our study, which used expert opinions, this research used a statistical algorithm to produce a shortened list. Furthermore, according to the Delphi evaluation by expert consensus in Iran, 15 items were identified, of which seven (house ownership, personal computer/laptop, smart cell phone, 3D TV, dishwasher, microwave, and car ownership) were asset ownership indicators [24]. Comparing results across countries is challenging because indicator selection, construction approaches, and item sets differ.

To our knowledge, no study has used an expert-opinion approach to select DHS variables for measuring SES in Cameroon. Our study involved experts from diverse backgrounds directly involved in the DHS, which strengthens the relevance of this expert-prioritization exercise to the national context. However, because this study did not construct or validate a composite index against an external SES or health-outcome standard, future work must formally establish the generalisability of any resulting index to national-level data and population subgroups.

Many studies have highlighted several limitations of the PCA approach. One notable limitation is "urban bias," as it relies on items that better reflect social stratification in urban areas than in rural areas. For example, urban households are much more likely to have access to electricity than rural households. In contrast, items rural households may have, such as access to land and livestock, are less often considered [10]. Furthermore, a study by Houweling et al. indicated that while PCA can provide a convenient way to construct composite indices, it may yield odd results when applied to short lists of items [25].

Expert opinion is essential when selecting indicators for socioeconomic measurement. When selecting indicators, experts consider factors such as cultural relevance, local context, and survey feasibility. Nevertheless, relying on expert opinion alone could introduce biases based on subjective judgment, personal experience, or limited perspectives [18].

In this context, experts can provide prior beliefs on the relationship between ownership indicators and SES. These prior beliefs can be formalized as Bayesian priors and combined with observed data on ownership indicators to update and refine SES estimates. Although the expert opinion approach is an innovative method of measuring SES, its underlying subjectivity can introduce bias, so it is important to balance expert opinion with empirical evidence. In support of this, our results recommend developing a Bayesian model that integrates expert opinion and empirical evidence to improve the robustness of SES measurement by providing more detailed insight into the socioeconomic context.

Limitations

Although the study provides a valuable contribution to the field, it is important to recognize its limitations. Like other opinion survey studies, our study relies on judgment and perception. Our study remains reliable because we reduced potential selection bias by obtaining a representative list of experts from the Cameroon National Institute of Statistics, and the questionnaire showed excellent internal consistency.

## Conclusions

This study presents a new approach to selecting DHS variables to measure SES and highlights the importance of expert input. We identified key indicators, particularly ownership of items such as refrigerators, televisions, laptops, and other household appliances deemed essential for accurately assessing populations’ socioeconomic status, particularly in Cameroon. This research underscores the importance of integrating local expert insights to refine SES measurement and promote improved health outcomes, particularly in Cameroon. Future research should explore applying this expert-opinion-driven framework in various contexts to develop more comprehensive, robust, and reliable SES indicators.

## Appendices

**Table 4:** Questionnaire on "estimating socioeconomic status for health equity surveillance in Cameroon: an expert opinion survey"

INTRODUCTION: Dear participant, my name is Buh Collins, and I am a researcher in the Department of Public Health, Faculty of Medicine and Biomedical Sciences, University of Yaoundé 1. I am undertaking a study to estimate socioeconomic status (SES) for health equity surveillance in Cameroon using variables from the Demographic and Health Survey (DHS). This study aims to identify the DHS assets most relevant for measuring SES in Cameroon, based on expert opinion. The DHS uses 29 household durable-goods indicators to construct the wealth index. In this study, we want to assess your expert opinion on whether you agree that each of the 29 DHS household asset indicators is a useful measure of SES. Your participation in this interview is voluntary but very important. Your answers will be kept strictly confidential. We will ensure that any statements or comments you make are not linked to your organization or to you as an individual.						
CONSENT TO PARTICIPATE: Do you agree to take part in this study? ☐ YES - Please continue ☐ NO - Thank you; the interview ends here						
SECTION A - RESPONDENT IDENTIFICATION						
1	Age: (_____) years					
2	Sex: ☐ Male ☐ Female					
3	Marital status: ☐ Married ☐ Cohabiting ☐ Divorced/separated ☐ Widowed ☐ Never married					
4	Basic training received: ☐ Medicine ☐ Public health ☐ Pharmacy ☐ Statistics ☐ Biology ☐ Social sciences ☐ Economics ☐ Demography ☐ Other: _______________					
5	Level of education: ☐ Bachelor ☐ Masters ☐ Doctorate ☐ Other: _______________					
6	Years of professional experience: (_____) years					
7	Type of institution: ☐ University ☐ NGO ☐ Research institute ☐ Government programme ☐ Other: _______________					
8a	If researcher, grade: ☐ Research associate ☐ Research fellow ☐ Research master ☐ Research director ☐ Other: _______________					
8b	If teacher-researcher: ☐ Assistant ☐ Lecturer ☐ Senior lecturer ☐ Professor ☐ Other: _______________					
SECTION B - KNOWLEDGE ABOUT THE DHS						
9	Have you ever heard of the DHS?					☐ Yes ☐ No
10	Do you have access to DHS data?					☐ Yes ☐ No
11	Have you been trained to use the DHS?					☐ Yes ☐ No
11a	If yes, where did you receive training? __________________________________________________					
12	Have you ever used DHS data?					☐ Yes ☐ No
13	Are you often involved in DHS design?					☐ Yes ☐ No
14	Are you often involved in analyzing DHS data?					☐ Yes ☐ No
15	Have you ever been involved in a DHS survey?					☐ Yes ☐ No
SECTION C - PERCEPTION OF SES INDICATORS						
16. In your opinion, which of the following 29 DHS household asset indicators are useful measures of socioeconomic status (SES)? Please rate each indicator on a scale from 1 to 5: (1 = strongly disagree, 2 = disagree, 3 = neither agree nor disagree, 4 = agree, 5 = strongly agree)						
#	Household asset indicator (DHS code)	1 strongly disagree	2 disagree	3 neither agree nor disagree	4 agree	5 strongly agree
1	Radio (HV207)					
2	Television (HV208)					
3	Home phone (HV221)					
4	Desktop computer (HV243E)					
5	Refrigerator (HV209)					
6	Stove (SH121G)					
7	Gas stove (SH121H)					
8	Air conditioner (SH121I)					
9	Fan (SH121J)					
10	CD/DVD player (SH121K)					
11	Grinding mill (SH121L)					
12	Mixer/grinder (SH121M)					
13	Modem/Internet key (SH121N)					
14	Cable/satellite (SH121O)					
15	Generator (SH121OP)					
16	Solar panel (SH121Q)					
17	Water pump (SH121R)					
18	Clock/pendulum (SH121S)					
19	Watch (HV243B)					
20	Cell phone (HV243A)					
21	Bicycle (HV210)					
22	Motorcycle or scooter (HV211)					
23	Animal-drawn cart (HV243C)					
24	Car or truck (HV212)					
25	Motorboat (HV243D)					
26	Laptop/notebook (SH122H)					
27	Computer tablet (SH122I)					
28	Owns land usable for agriculture (HV244)					
29	Owns livestock, herds, or farm animals (HV246)					
